# Author Correction: Plastic waste discharge to the global ocean constrained by seawater observations

**DOI:** 10.1038/s41467-023-43910-y

**Published:** 2023-12-18

**Authors:** Yanxu Zhang, Peipei Wu, Ruochong Xu, Xuantong Wang, Lili Lei, Amina T. Schartup, Yiming Peng, Qiaotong Pang, Xinle Wang, Lei Mai, Ruwei Wang, Huan Liu, Xiaotong Wang, Arjen Luijendijk, Eric Chassignet, Xiaobiao Xu, Huizhong Shen, Shuxiu Zheng, Eddy Y. Zeng

**Affiliations:** 1 School of Atmospheric Sciences, Nanjing University, 210023 Nanjing, China; 2 Frontiers Science Center for Critical Earth Material Cycling, Nanjing University, 210023 Nanjing, China; 3 Scripps Institution of Oceanography, University of California, San Diego, La Jolla, CA USA; 4 Center for Environmental Microplastics Studies, Guangdong Key Laboratory of Environmental Pollution and Health, School of Environment, Jinan University, 511443 Guangzhou, China; 5 State Key Joint Laboratory of ESPC, State Environmental Protection Key Laboratory of Sources and Control of Air Pollution Complex, School of Environment, Tsinghua University, Beijing, China; 6 Faculty of Civil Engineering and Geosciences, Delft University of Technology, Delft, Netherlands; 7 Hydraulic Engineering, Deltares, Delft, Netherlands; 8 Center for Ocean–Atmospheric Prediction Studies (COAPS), Florida State University, Tallahassee, FL USA; 9 School of Environmental Science and Technology, Southern University of Science and Technology, Shenzhen, Guangdong China; 10 College of Urban and Environmental Sciences, Peking University, Beijing, China

**Keywords:** Ocean sciences, Environmental sciences

Correction to: *Nature Communications* 10.1038/s41467-023-37108-5, published online 13 March 2023

The original version of this Article contained an error in Fig. 2, in which c was incorrectly described as “middle scenario” where it should have been described as “low scenario”. The correct version of Fig. 2 is:
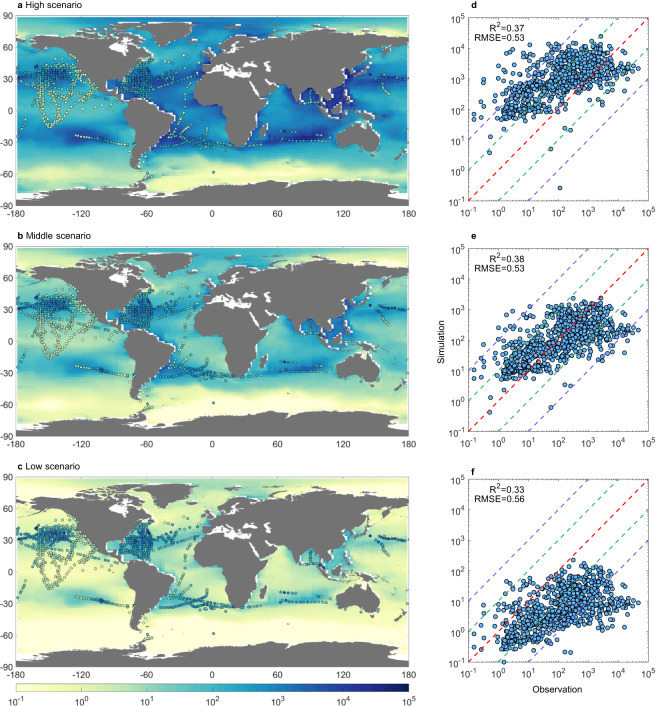


which replaces the previous incorrect version:
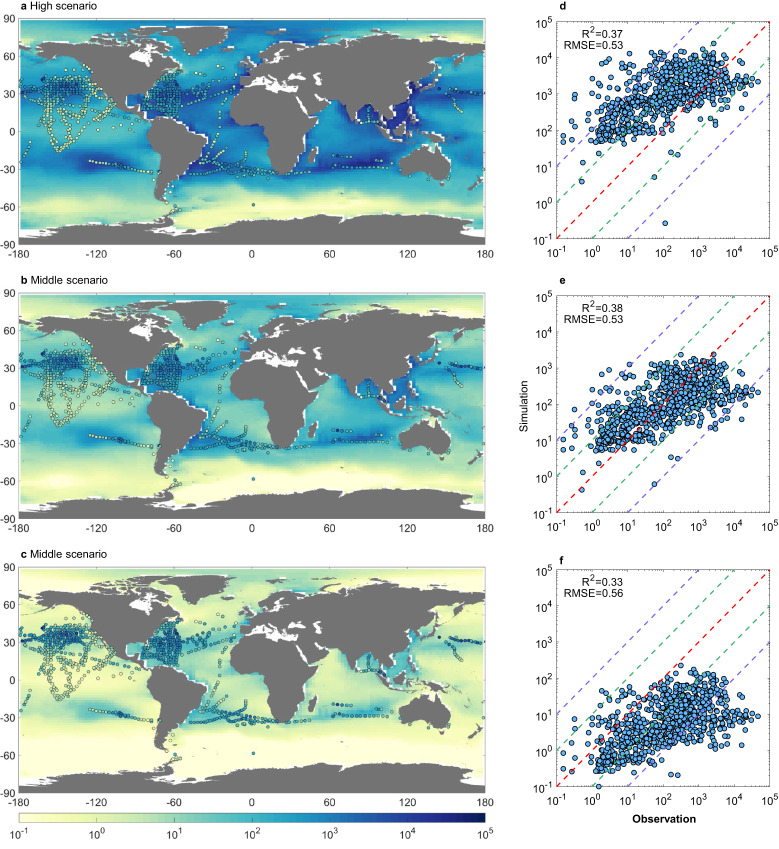


This has been corrected in both the PDF and HTML versions of the Article.

