# Supplementary material for: Plastic waste discharge to the global ocean constrained by seawater observations
Source: Nat Commun. 2023 Mar 13;14:1372. doi: 10.1038/s41467-023-37108-5 (PMC10011382; doi:10.1038/s41467-023-37108-5)
Supplement: Supplementary file 1 — Supplementary Information [file 41467_2023_37108_MOESM1_ESM.pdf]

Supplementary Materials for

Plastic waste discharge to the global ocean constrained by seawater observations

Yanxu Zhang[1,2*†], Peipei Wu[1†], Ruochong Xu[1], Xuantong Wang[1], Lili Lei[1*], Amina T. Schartup[3], Yiming Peng[1], Qiaotong Pang[1], Xinle Wang[1], Lei Mai[4], Ruwei Wang[4], Huan Liu[5], Xiaotong Wang[5], Arjen Luijendijk[6,7], Eric Chassignet[8], Xiaobiao Xu[8], Huizhong Shen[9], Shuxiu Zheng[10], Eddy Y. Zeng[4*]

[1]School of Atmospheric Sciences, Nanjing University; Nanjing 210023, China.

[2]Frontiers Science Center for Critical Earth Material Cycling, Nanjing University; Nanjing 210023, China

[3]Scripps Institution of Oceanography, University of California, San Diego; La Jolla, CA, USA.

[4]Center for Environmental Microplastics Studies, Guangdong Key Laboratory of Environmental Pollution and Health, School of Environment, Jinan University; Guangzhou 511443, China.

[5]State Key Joint Laboratory of ESPC, State Environmental Protection Key Laboratory of Sources and Control of Air Pollution Complex, School of Environment, Tsinghua University; Beijing, China.

[6]Faculty of Civil Engineering and Geosciences, Delft University of Technology; Delft, Netherlands.

[7]Hydraulic Engineering; Deltares, Delft, Netherlands.

[8]Center for Ocean-Atmospheric Prediction Studies (COAPS), Florida State University; Tallahassee, FL, United States.

[9]School of Environmental Science and Technology, Southern University of Science and Technology; Shenzhen, Guangdong, China.

[10]College of Urban and Environmental Sciences, Peking University; Beijing, China

[*]Corresponding author. Email: YZ zhangyx@nju.edu.cn; LL lililei@nju.edu.cn; and EYZ eddyzeng@jnu.edu.cn

[†]These authors contributed equally.

Supplementary Discussion

Inclusion of seawater observations

Supplementary Table 8 lists the observed surface ocean plastic abundance data used in this study. Isobe et al. compiled a multilevel dataset that includes almost all available ocean plastic abundance data so far[1]. The Isobe database includes a total of 9959 data points obtained by Neuston net, WP2 net, Manta net, Bango net, and Plankton net. The identification method includes visual identification, FTIR (Fourier transform infrared spectrophotometer), and Raman spectroscopy. There is a large discrepancy among data obtained with a different method[2,3]. The risk of clogging and uncertainty in sample volumes also depends on the mesh size of the net and the flow conditions[3]. Visual inspection is known to lead to an overestimation of the particle count because 20-70% of the particles identified visually as plastics might be of other chemical compositions, e.g., coal ash, especially for the particle smaller than 500 µm[4,5]. However, visual identification misses the transparent and small microplastics thus underestimating the plastic concentrations[2]. The data obtained by different methodologies are thus generally not comparable with each other. More importantly, different methodologies are used in different studies covering different ocean basins. A mixture of data obtained by different methodologies may distort the real spatial pattern of surface ocean plastic abundance and reduce the reliability of the comparison between simulations and observations. There is a trade-off between "more data" and "ghost spatial pattern" arising from different measurement methodologies. Therefore, we use only the dataset obtained by Neuston net and the visual identification method due to its large sampling number and spatial coverage. The number of such data is 7431 (Supplementary Table 8), accounting for 75% of the Isobe database. The data in different ocean basins are also comparable due to similar methodology.

All the data used in this study are reported as numerical concentrations (*N*), which are transferred to mass concentrations (*M*) following Cozar et al.[6]:

$$\log M(g \cdot km^{-2}) = 1.21 \times \log N(items \cdot km^{-2}) - 3.99 \quad (1)$$

and are compared with modeled mass concentrations of plastics in the surface ocean. Such a transfer does not change the number of independent measurements used in this study. The conversion bears uncertainty but the uncertainty is reduced due to the large sampling number[7]. The item-mass conversion factors of all observed concentrations range from 1.3×10^{-5} to 2.4×10^{3} g per item, with a mean value of 31 g per item. However, mass concentrations (or total mass) are more comparable with plastic emission inventories that are also in a mass unit. Moreover, the modeled number concentrations are prone to larger uncertainties due to the fragmentation process, which is mass-conservative but could greatly change the number concentrations[6].

Supplementary Methods

Other sources of plastics to the ocean than MPW

There are other potential sources contributing to the ocean plastics beyond riverine discharge, coastal erosions, and marine sources that are considered in this study. One potential source is atmospheric transport and deposition. The total deposition of plastics to ocean surfaces from land sources is 13 kilo Mt yr^{-1}, much smaller than the three sources mentioned in the main text. Moreover, there is a suggested net transport of 9 kilo Mt yr^{-1} plastics from ocean to land, i.e. the loss of sea surface ocean plastics to the atmosphere (via mechanical processes similar to sea salt

emissions) surpasses that is deposited to the ocean[8]. Another source is direct wastewater to ocean by coastal population (other wastewater enters the ocean via rivers, hence being considered in this study), which is estimated about 0.44 kilo Mt yr^{-1}, even smaller than what we already considered[9]. Other plastic sources than mismanaged plastic waste include unintentional plastic emissions, such as fibers from textiles, tire wear particles, and lost resin pellets. We assume that they have been included in the riverine inventories of plastic waste discharge, as these inventories were built upon riverine measurements that cover all plastic types. Natural disasters are also potential significant sources. For example, the Fukishima earthquake and tsunami in March, 2011 released ~20 million Mt of debris to the ocean[10]. However, the emission of plastic waste in such events is poorly quantified at global scale, and its contribution to the ocean plastics is rather small given its unusual and episodic nature.

Stokes drift

We take the estimated Stokes drift velocity from the GlobCurrent and apply it to all the plastic tracers in our model[11]. The data are available between 1990 and 2015, with a time resolution of 3 hours. We calculate a 26-year average of the Stokes drift velocity for each month. In this way, 12 months of Stokes drift velocity is achieved and cycled in the model for the whole simulation period. The strongest Stokes drift is simulated in the Southern Ocean and the high-latitude ocean in the northern hemisphere where plastic concentrations are relatively low (Supplementary Fig. 6), consistent with previous results[12]. The modeled plastic accumulation in the subtropical gyres is moved westward slightly by Stokes drift, while plastics in the Southern Ocean are moved eastward.

Sinking and rising

The sinking/rising rate of plastics depends on its density. Plastic particles in our model are treated as spheres. At steady state, the forces acting on plastic particles are balanced:

$$\mathbf{F_D} + \mathbf{F_g} + \mathbf{F_b} = 0 \tag{2}$$

where $\mathbf{F_D}$ is vertical dragging force, $\mathbf{F_g}$ is gravity, and $\mathbf{F_b}$ is buoyancy. These forces are calculated as:

$$\mathbf{F_g} = V_p \rho_p \mathbf{g} \tag{3}$$

$$\mathbf{F_b} = -V_s \rho_s \mathbf{g} \tag{4}$$

$$\mathbf{F_D} = -\frac{1}{2} C_D(\mathrm{Re}_s) A_p \rho_s \frac{(\mathbf{w} - \mathbf{w_s})^3}{|\mathbf{w} - \mathbf{w_s}|} \tag{5}$$

where V_p is the volume of the particle, while V_s is the volume of the particle that is submerged in seawater ($V_p = V_s$ in this case, but $V_p > V_s$ for floating particles with zero sinking/rising velocity relative to the seawater, e.g., unbiofouled PP and PE). C_D is the coefficient of dragging, which is a function of the Reynolds number (Re) of a certain motion of a fluid. A_p is the horizontal sectional area of a particle, ρ_s is the density of seawater, ρ_p is the mean density of a particle, $\mathbf{w}$ is the vertical velocity of the particle, $\mathbf{w_s}$ is the vertical velocity of seawater, and $\mathbf{g}$ is the gravity acceleration.

Based on Supplementary Equation (2)–(5), we get $(\mathbf{w}\text{-}\mathbf{w_s})^2$:

$$(\mathbf{w} - \mathbf{w}_s)^2 = \frac{4|\mathbf{g}|d(\rho_p - \rho_s)}{3C_D(\mathrm{Re}_s)\rho_s} \tag{6}$$

where d is Stokes diameter of a particle, and C_D is calculated as[13]:

$$C_D(\mathrm{Re}) = \begin{cases} 24\,\mathrm{Re}^{-1} & \mathrm{Re} \le 0.3 \\ 18.5\,\mathrm{Re}^{-0.6} & 0.3 < \mathrm{Re} \le 1000 \\ 0.44 & 1000 < \mathrm{Re} \le 20000 \end{cases} \tag{7}$$

Re_s is the Re of seawater and is calculated as:

$$\mathrm{Re}_s = \frac{d\rho_s |\mathbf{w} - \mathbf{u_s}|}{\mu_s} \tag{8}$$

Based on Supplementary Equation (6) (7), we get $\mathbf{w}$-$\mathbf{w_s}$:

$$\mathbf{w} - \mathbf{w}_s = \begin{cases} \dfrac{d^2 g(\rho_p - \rho_s)}{18\mu} & \mathrm{Re} \le 0.3 \\ \dfrac{0.153 d^{1.143}[g(\rho_p - \rho_s)]^{0.714}}{\rho_s^{0.286}\mu^{0.429}} & 0.3 < \mathrm{Re} \le 1000 \\ 1.74\left(\dfrac{dg(\rho_p - \rho_s)}{\rho_s}\right)^{\frac{1}{2}} & 1000 < \mathrm{Re} < 200000 \end{cases} \tag{9}$$

Supplementary Equation (6) is a piecewise function. The three conditions of different Re are all possible in reality because of the variety of particles, but it is hard to know which range of Supplementary Equation (6) should be used in a certain situation, since Re should be calculated directly by sinking velocity $\mathbf{w}$, which is unknown here.

Notice that Supplementary Equation (7) can be represented by:

$$C_D = \frac{a}{\mathrm{Re}^b} \quad \begin{cases} a = 24, b = 1 & \mathrm{Re} \le 0.3 \\ a = 18.5, b = 0.6 & 0.3 < \mathrm{Re} \le 1000 \\ a = 0.4, b = 0 & 1000 < \mathrm{Re} < 200000 \end{cases} \tag{10}$$

Combine Supplementary Equation (6) (8) (10) and eliminate $|\mathbf{w}$-$\mathbf{u_s}|$ and C_D:

$$\mathrm{Re} = \left(\frac{4}{3a}\right)^{\frac{1}{2-b}} \left(d^3 \frac{\rho_s g(\rho_p - \rho_s)}{\mu^2}\right)^{\frac{1}{2-b}} \tag{11}$$

Define K:

$$K = d^3 \frac{\rho_s g(\rho_p - \rho_s)}{\mu^2} \tag{12}$$

Then:

$$\mathrm{Re} = \begin{cases} 0.0556K & \mathrm{Re} \le 0.3 \\ 0.072K^{\frac{5}{7}} & 0.3 < \mathrm{Re} \le 1000 \\ 1.74K^{\frac{1}{2}} & 1000 < \mathrm{Re} < 200000 \end{cases} \tag{13}$$

$$\mathbf{w}-\mathbf{w}_s=\begin{cases}\dfrac{d^2 g(\rho_p-\rho_s)}{18\mu} & K\le 5.4\\ \dfrac{0.153 d^{1.143}[g(\rho_p-\rho_s)]^{0.714}}{\rho_s^{0.286}\mu^{0.429}} & 5.4<K\le 24\\ 1.74\left(\dfrac{dg(\rho_p-\rho_s)}{\rho_s}\right)^{\frac{1}{2}} & K>24\end{cases} \tag{14}$$

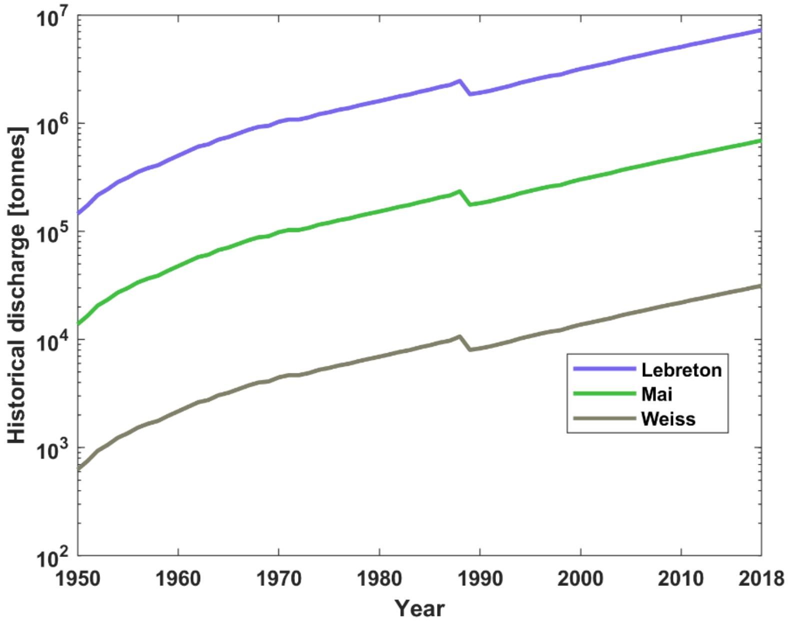

Supplementary Fig. 1: The historical trends of global total plastic emissions during 1950-2018 for the High (Lebreton), Middle (Mai), and Low (Weiss) scenarios. The abrupt decrease in the 1980s is associated with the MARPOL Convention that bans the damping of waste from ships.

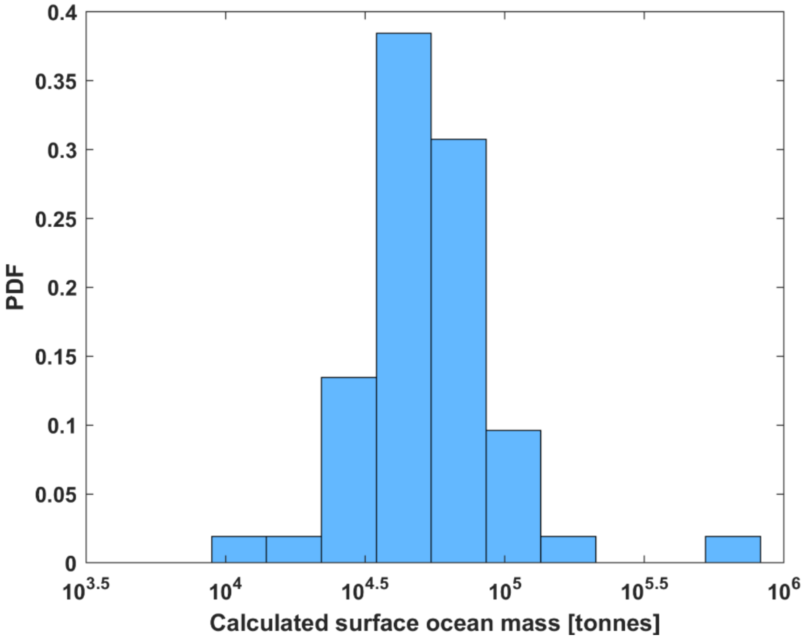

Supplementary Fig. 2: Probability distribution function of the calculated surface ocean plastic mass. The results are from the ensemble members ($N = 52$) driven by the Middle emission scenario.

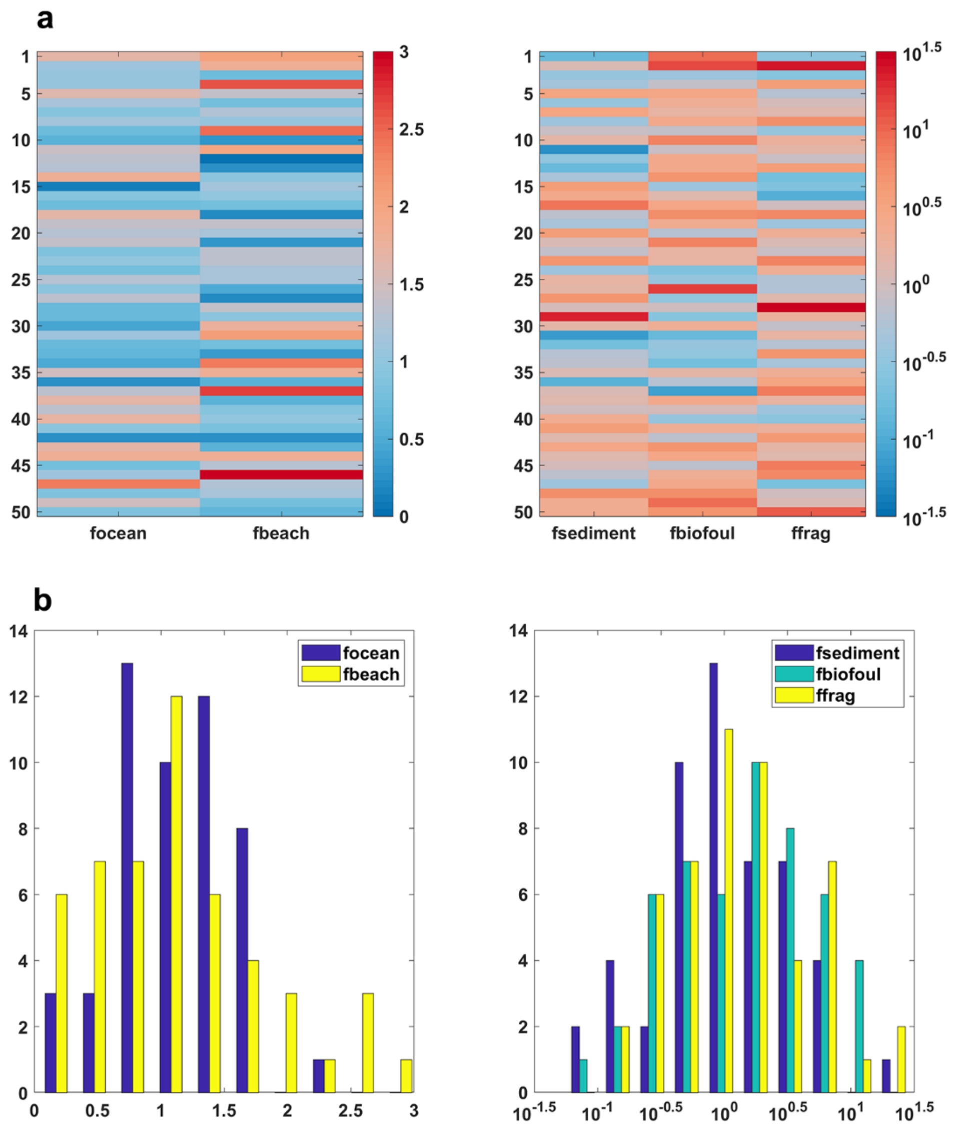

Supplementary Fig. 3: Parameters of the model ensemble (*N* = 50). a parameter values. **b** frequency distributions of the values. Parameters are represented as the ratio to the corresponding values in the test case simulation (Table 1), including focean: fraction of marine discharge to the total discharge; fbeach: beaching rate; fsediment: sedimentation rate; fbiofoul: biofouling rate; ffrag: fragmentation rate.

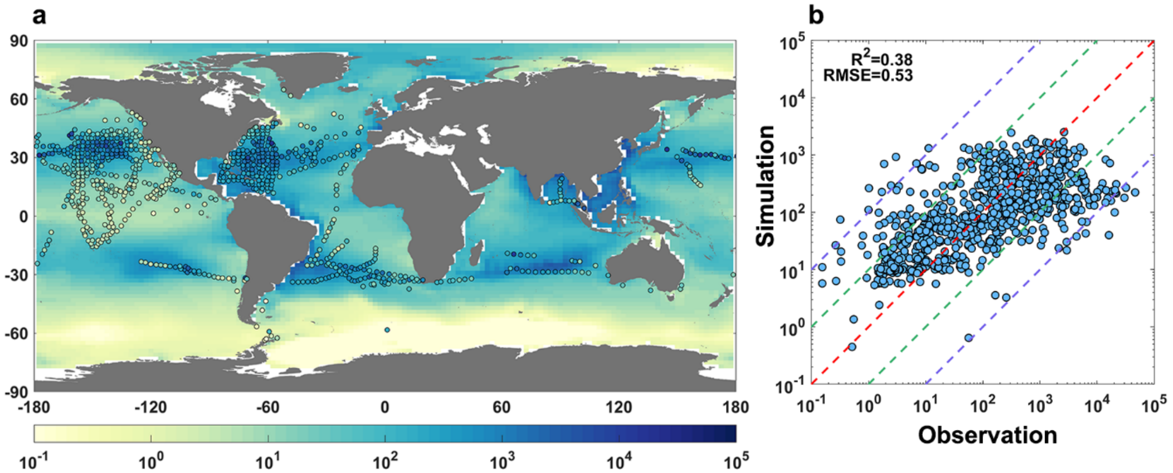

Supplementary Fig. 4: Comparison between observed and modeled surface ocean plastic mass concentrations driven by the best-estimate emissions. The background colors in panel **a** are the modeled results, while the circles are observations. The dashed lines in panel **b** are 100:1, 10:1, 1:1, 1:10, and 1:100. All the concentrations, and the calculation of R^2 and RMSE are in a base 10 logarithmic scale with a unit of g km^{-2}. The best-estimate emission is close to the middle emission scenario and the simulated concentrations in panel **b** are just slightly higher than those in Fig. 2e.

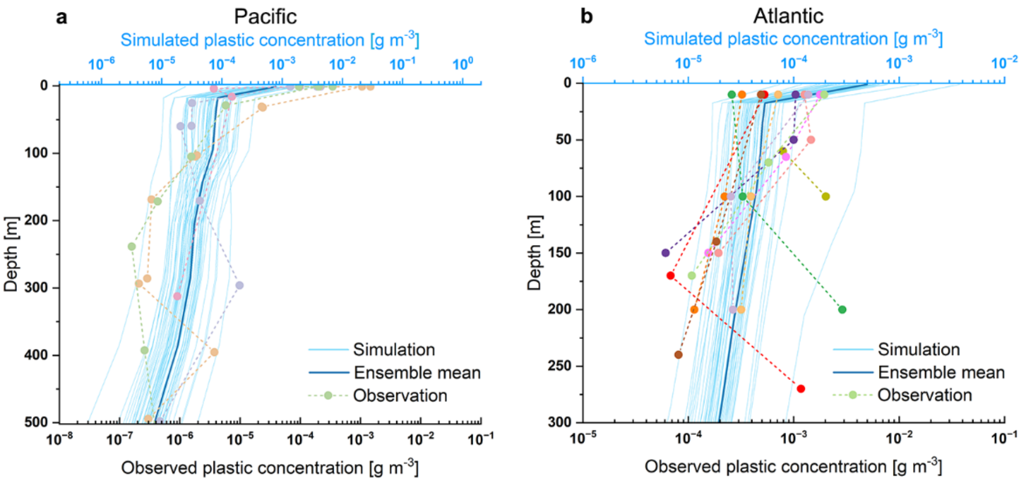

Supplementary Fig. 5: Comparison between observed and modeled vertical profile of plastic mass. **a** Pacific. **b** Atlantic Ocean. The dash lines indicate individual observed profiles with the filled-colored circles representing sampling depths. The light blue solid lines are the mean plastic mass concentrations over the sampling sites by the 52 member models under the middle emission scenario (other emission scenarios simulate higher or lower concentrations but with similar vertical trends), while the dark blue solid lines are the ensemble means. The observations are from Egger et al. (2020) and Pabortsave and Lampitt (2020) for the Pacific and Atlantic Ocean, respectively[14,15].

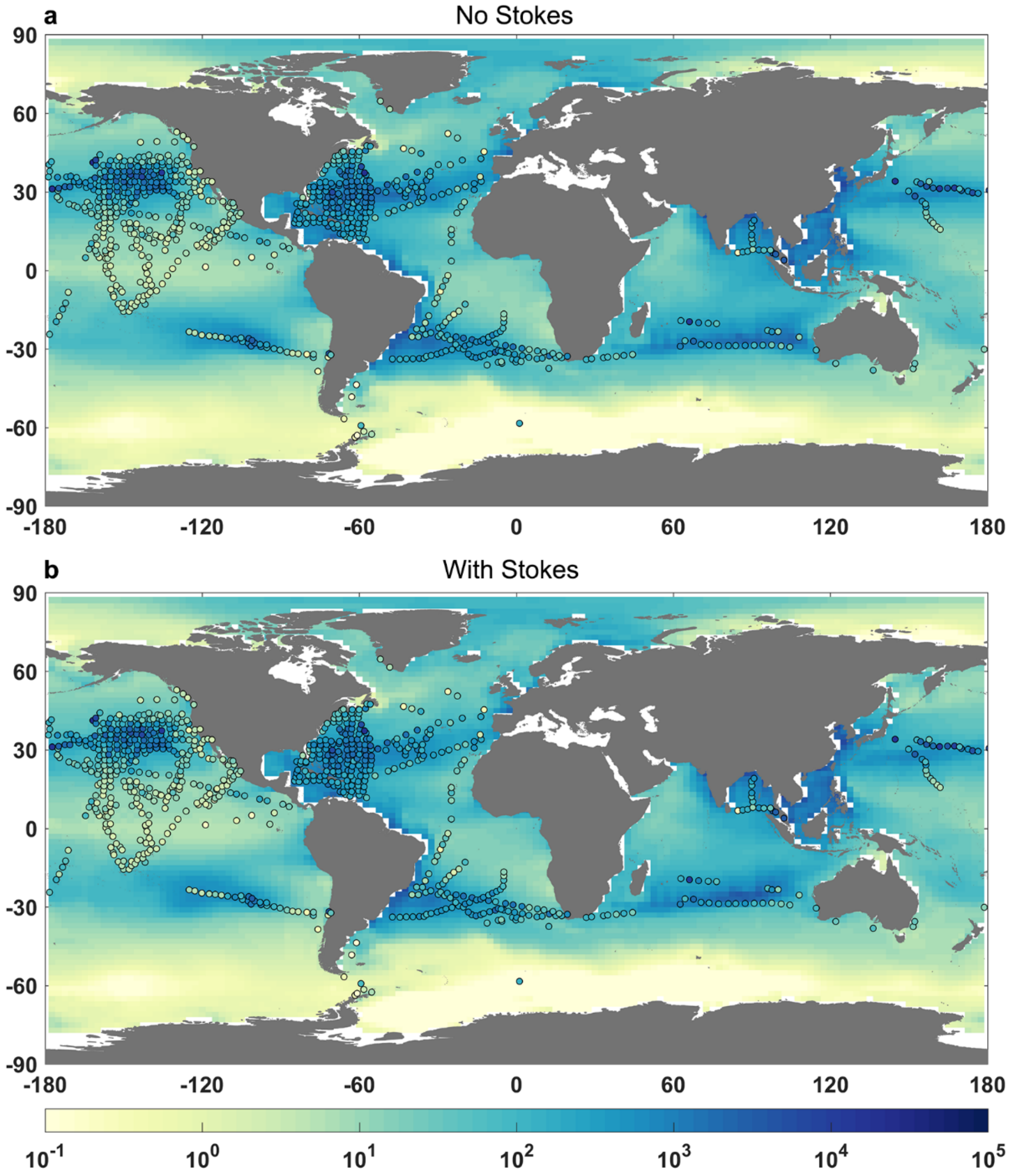

Supplementary Fig. 6: The modeled spatial pattern of surface plastic abundance. a Without Stokes drift. **b** With Stokes drift. Compared with panel **a**, Stokes drift slightly changes the spatial pattern in panel **b**.

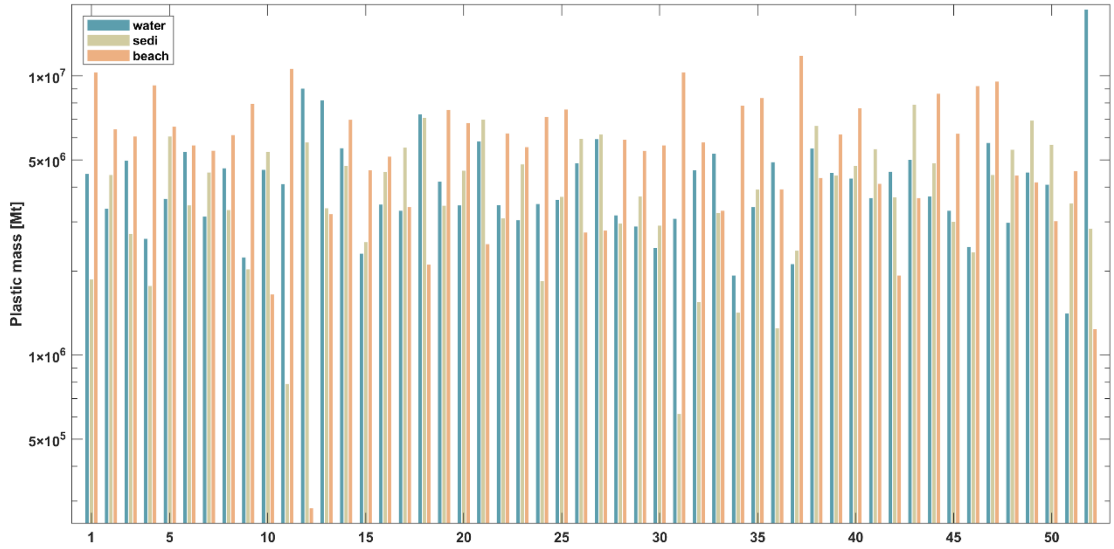

Supplementary Fig. 7: Modeled amount of plastics in the water column, sediments, and beaches. The 52 ensemble members are driven by the middle emission scenario. The parameters of the model members are listed in Supplementary Table 7.

Supplementary Table 1. Mapping of emission inventory to the size bins of our model

Emission inventory	Model tracers
Microplastics	All assumed as the <0.0781-5 mm bin
Macroplastics	Equally distributed between 5-50 mm and > 50 mm bins

Supplementary Table 2. Fraction of emissions from different continents

	Lebreton	Mai	Weiss
Asia	86.00%	69.00%	45.34%
Africa	7.80%	1.55%	11.31%
North America	0.95%	6.23%	14.78%
South America	4.80%	21.39%	17.31%
Europe	0.28%	1.71%	10.61%
Oceania	0.02%	0.16%	0.65%

Supplementary Table 3. Consumption of different plastic types around the world in 2013, in the unit of 10 thousand metric tons[16].

Type	Global	Asia	North America	Western Europe	Middle East
PE	8157.8	3648.1	1557.9	1101.2	534.5
PP	5606.8	3070.9	720.1	737.4	375.2
PVC	3854.9	2192.4	514.4	402	215.9
Others	2387.3	1345.9	323.8	326.1	126

Supplementary Table 4. Model parameterization of plastic particle settling.

Parameter	Unit	Description	Value or formula
$\mathbf{F_D}$	kg m s^{-2}	Dragging force of seawater (vertical)	$\mathbf{F_D} = -\frac{1}{2} C_D(\mathrm{Re}_s) A_p \rho_s \frac{(\mathbf{w}-\mathbf{w_s})}{\lvert \mathbf{w}-\mathbf{w_s} \rvert}$
$\mathbf{F_g}$	kg m s^{-2}	Gravity	$\mathbf{F_g} = V_p \rho_p \mathbf{g}$
$\mathbf{F_b}$	kg m s^{-2}	Buoyancy	$\mathbf{F_b} = -V_s \rho_s \mathbf{g}$
C_D	unitless	Coefficient of dragging (function of Re)	$C_D = C_D(\mathrm{Re})$
Re_s	unitless	Reynolds number of seawater	$\mathrm{Re}_s = \frac{d \rho_s \lvert \mathbf{w}-\mathbf{w_s} \rvert}{\mu_s}$
A_p	m^2	Sectional area	$A_p = \frac{1}{4} \pi d^2$
d	m	Stokes diameter (For strict sphere particle, stokes diameter is normal diameter. For other shapes of particles, stokes diameter is the diameter of the sphere particle which has the same sinking speed.)	Model input
V_p	m^3	Volume of microplastics particle	$V_p = \frac{1}{6} \pi d^3$
Vs	m^3	Volume of microplastics particle submerged in sweater (when sinking or rising)	$Vs=V_p$
ρ_p	kg m^{-3}	Mean density of particle	Model input
ρ_s	kg m^{-3}	Density of sea water	Model input
$\mathbf{g}$	m s^{-2}	Gravitational acceleration	9.8
μ_s	kg m^{-1} s^{-1}	Dynamic-viscosity coefficient of seawater	Model input
$\mathbf{w}$	m s^{-1}	Sinking or rising speed (Need also a technique to eliminate Re. Flux of particle and settling of particle also have parameterization)	$(\mathbf{w}-\mathbf{w_s})^2 = \frac{4 \lvert \mathbf{g} \rvert d (\rho_p - \rho_s)}{3 C_D(\mathrm{Re}_s) \rho_s}$
$\mathbf{w_s}$	m s^{-1}	Velocity of seawater	Model input

Supplementary Table 5. Model parameterization of plastic particle drifting.

Parameter	Unit	Description	Value or formula
$\mathbf{F_s}$	kg m s^{-2}	Dragging force by sea water (horizontal)	$\mathbf{F_s} = -\frac{1}{2} C_D(\mathrm{Re}_s) A_s \rho_s \frac{(\mathbf{u}-\mathbf{u_s})^3}{\lvert \mathbf{u}-\mathbf{u_s} \rvert}$
$\mathbf{F_a}$	kg m s^{-2}	Dragging force by air (horizontal)	$\mathbf{F_a} = -\frac{1}{2} C_D(\mathrm{Re}_a) A_a \rho_a \frac{(\mathbf{u}-\mathbf{u_a})^3}{\lvert \mathbf{u}-\mathbf{u_a} \rvert}$
$\mathbf{F_c}$	kg m s^{-2}	Coriolis force	$\mathbf{F_c} = V_p \rho_p f_C \mathbf{u}$
Re_a	unitless	Reynolds number of seawater	$Re_a = \frac{d \rho_a \lvert \mathbf{u}-\mathbf{u_a} \rvert}{\mu_a}$
Vs	m^3	Volume of microplastics particle submerged in sweater	$V_s = \frac{1}{6}\pi d^3 - \pi h^2 (\frac{d}{2} - \frac{h_a}{3})$
h_a	m	height of the upper part (exposed to air)	By solving $\mathbf{F_g} + \mathbf{F_b} = 0$
A_a	m^2	Sectional area of particle exposed to air	$A_a = \frac{1}{4} r^2 (\alpha - \sin\alpha)$<br>$\alpha = 2 \arccos(1 - \frac{2h_a}{d})$
A_s	m^2	Sectional area of particle exposed to seawater	$A_s = \frac{1}{4}\pi d^2 - A_a$
ρ_a	kg m^{-3}	Density of air	Model input
f_C	rad s^{-1}	Coriolis parameter	$f_C = 2\Omega \sin\varphi$
Ω	rad s^{-1}	Angular speed of the earth	7.3×10^{-5}
φ	degree	Latitude	Model grid
$\mathbf{u}$	m s^{-1}	Drifting speed (using gradient descent and fourth-order Adams method to calculate)	$m\frac{d\mathbf{u}}{dt} = \mathbf{F_s} + \mathbf{F_a} + \mathbf{F_c}$

Supplementary Table 6. Model parameterization of plastic particle biofouling and defouling[17].

Parameter	Unit	Description	Value or formula
V_{bf}	m^3	Volume of biofilm	$\frac{dV_{bf}}{dt} = V_a \theta_p \frac{dA}{dt} + V_a A \frac{d\theta_p}{dt}$
V_a	m^3	Individual algae volume	2.0×10^{-16}
A	# m^{-2}	the biomass of attached algae	$\frac{dA}{dt} = \frac{\beta_a A_a}{\theta_p} - m_a A$
θ_p	m^2	surface area of plastic particle	Model input
β_a	m^3 s^{-1}	Encounter kernel rate	$\beta_a = \beta_{brownian} + \beta_{shear}$
A_a	# m^{-3}	Ambient algae concentration	Model input
m_a	s^{-1}	Mortality rate	4.5×10^{-6}
$\beta_{brownian}$	m^3 s^{-1}	Brownian motion frequencies	$\beta_{brownian} = 4\pi(D_p + D_a)(r_p + r_a)$
β_{shear}	m^3 s^{-1}	Advective shear collision frequencies	$\beta_{shear} = 1.3\gamma(r_p + r_a)^3$
D_p	m^2 s^{-1}	Diffusivity of plastics	$D_p = \frac{k(T + 273.16)}{6\pi\mu_{sw} r_p}$
D_a	m^2 s^{-1}	Diffusivity of individual algae cells	$D_a = \frac{k(T + 273.16)}{6\pi\mu_{sw} r_a}$
r_p	m	radius of plastics	Model input
r_a	m	radius of algae cells	Model input
k	kg m^{-1} s^{-1}	Boltzmann constant	1.3806×10^{-23}
T	°C	Seawater temperature	Model input
ρ_{bf}	kg m^{-3}	Algae density	1388
γ	s^{-1}	Shear rate	1.9676
μ_{sw}	kg m^{-1} s^{-1}	Dynamic water viscosity	1.174×10^{-3}
τ_{trans}	s^{-1}	Fraction of transformation between biofouled and unbiofouled plastics	$\tau_{trans} = \delta \frac{\frac{dV_{bf}}{dt}}{\Delta V}$
ΔV	m^3	Deviation of volume between the two plastics	Calculate in the model
$V_{PE_{neutral}}$	m^3	Volume of a neutral PE particle	$\frac{4}{3}\pi r^3_{PE_{neutral}}$
$V_{PE_{floating}}$	m^3	Volume of a floating PE particle	$\frac{4}{3}\pi r^3_{PE_{floating}}$

Supplementary Table 7. Parameters of ensemble model members used in this study (ratios to those of the test scenario).

No.	focean[a]	fbeach[b]	fsediment[c]	fbiofoul[d]	ffrag[e]
**1**	1.65	2.06	0.15	9.37	0.29
**2**	1.11	1.82	1.18	14.06	23.78
**3**	1.07	0.73	0.38	0.41	0.27
**4**	1.04	2.58	0.44	0.72	3.83
**5**	1.61	1.36	3.26	2.72	0.64
**6**	1.21	0.78	0.35	2.08	1.08
**7**	0.91	1.22	3.12	1.57	1.25
**8**	1.14	1.04	0.41	2.47	5.06
**9**	0.73	2.45	0.82	0.76	0.36
**10**	0.60	0.29	1.50	6.29	1.84
**11**	1.43	2.01	0.06	0.82	1.50
**12**	1.36	0.02	0.31	2.49	0.87
**13**	1.28	0.28	0.15	2.40	3.95
**14**	1.82	0.97	0.49	4.54	0.19
**15**	0.13	1.15	3.95	0.40	0.23
**16**	0.93	1.01	2.60	1.28	0.11
**17**	0.73	0.77	8.07	2.87	0.94
**18**	1.65	0.22	0.67	5.08	5.64
**19**	1.37	1.37	0.51	2.36	0.39
**20**	1.29	1.21	3.71	0.61	2.08
**21**	1.40	0.33	1.20	6.29	1.14
**22**	0.85	1.34	0.73	1.67	0.86
**23**	0.99	1.35	4.80	1.68	6.67
**24**	0.78	1.18	0.38	0.23	2.03
**25**	1.31	1.22	1.58	0.37	0.56
**26**	0.95	0.48	1.47	15.23	0.57
**27**	1.32	0.22	4.67	0.33	1.27
**28**	0.69	1.38	1.01	0.95	29.82
**29**	0.68	0.96	21.95	0.27	1.89
**30**	0.46	1.77	1.46	2.12	0.73
**31**	1.12	2.10	0.07	0.15	1.61
**32**	0.67	0.78	0.18	0.35	0.63
**33**	0.64	0.35	0.62	0.34	4.69
**34**	0.51	2.36	0.65	0.19	0.49
**35**	1.47	1.79	1.20	1.39	2.07
**36**	0.27	0.56	0.12	0.60	2.98
**37**	1.36	2.72	1.21	0.08	6.99
**38**	1.64	0.56	1.29	2.45	1.56
**39**	1.35	0.92	0.96	1.11	0.40
**40**	1.71	1.00	2.20	0.37	0.29
**41**	0.95	0.81	3.96	2.01	1.86
**42**	0.28	0.27	1.26	0.67	4.42
**43**	1.67	0.56	2.74	4.99	1.45

**44**	1.81	1.80	1.28	2.74	1.25
**45**	0.79	1.27	1.04	0.66	7.68
**46**	1.11	3.05	0.71	1.91	6.18
**47**	2.36	1.23	0.40	2.52	0.22
**48**	0.86	1.20	5.56	5.23	0.97
**49**	1.46	0.76	2.14	9.07	1.20
**50**	0.85	0.62	2.28	4.66	11.56
**51**	0.13	3.05	21.95	15.23	29.82
**52**	2.36	0.02	0.06	0.08	0.11
**mean**[f]	1.11	1.18	2.37	2.87	3.62
**stdev**[g]	0.50	0.77	4.28	3.70	6.53

[a]fractions of the ocean emission to the total discharge. [b]beaching rate. [c]sedimentation rate. [d]biofouling rate. [e]fragmentation rate. [f]the mean of all the members. [g]the standard deviation of the members.

Supplementary Table 8. summarizes the data sources and measurement procedures.

Area	Sampling method	Mesh size [mm]	Number of data	Without fiber (%)	Flowmeter	Identification method	Unit	Reference
Eastern N. Pacific	N[a]	0.335	2529	NR[b]	W/O[c]	V[e]	pieces/km^2	Law et al. 2014[18]
World's ocean	N	0.2	1943	100[f]	W[d]	V	pieces/km^2	Cozar et al. 2014[6]
World's ocean	N	0.33	679	100[g]	W/O	V	pieces/km^2	Eriksen et al. 2014[19]
Western N. Atlantic & Caribbean Sea[h]	N	0.335	2280	NR	W/O	V	pieces/km^2	Law et al. 2010[20]

[a]Neuston net, [b]Not recorded, [c,d]Without or with a flowmeter, [e]Visual identification, [f]Fibrous microplastics were discarded by this project, [g]The "vast majority" of collected microplastics were fragments, [h]Also includes macroplastics.

Supplementary References:

1. Isobe, A. *et al.* A multilevel dataset of microplastic abundance in the world's upper ocean and the Laurentian Great Lakes. *Microplastics and Nanoplastics* **1** (2021).
2. Song, Y. K. *et al.* A comparison of microscopic and spectroscopic identification methods for analysis of microplastics in environmental samples. *Marine Pollution Bulletin* **93**, 202-209 (2015).
3. Bai, M., Lin, Y., Hurley, R. R., Zhu, L. & Li, D. Controlling factors of microplastic riverine flux and implications for reliable monitoring strategy. *Environmental Science & Technology* **56**, 48–61 (2021).
4. Eriksen, M. *et al.* Microplastic pollution in the surface waters of the Laurentian Great Lakes. *Marine Pollution Bulletin* **77**, 177-182 (2013).
5. Hidalgo-Ruz, V., Gutow, L., Thompson, R. C. & Thiel, M. Microplastics in the marine environment: A review of the methods used for identification and quantification. *Environmental Science & Technology* **46**, 3060–3075 (2012).
6. Cozar, A. *et al.* Plastic debris in the open ocean. *Proceedings of the National Academy of Sciences* **111**, 10239-10244, doi:10.1073/pnas.1314705111 (2014).
7. Roebroek, C. T. J., Laufkötter, C., González-Fernández, D. & van Emmerik, T. The quest for the missing plastics: Large uncertainties in river plastic export into the sea. *Environmental Pollution* **312** (2022).
8. Brahney, J. *et al.* Constraining the atmospheric limb of the plastic cycle. *Proceedings of the National Academy of Sciences* **118** (2021).
9. Weiss, L. *et al.* The missing ocean plastic sink: Gone with the rivers. *Science* **373**, 107-111 (2021).
10. BBC NEWS. *Japan's tsunami debris: Five remarkable stories*, <https://www.bbc.com/news/world-asia-35638091> (2016).
11. GlobCurrent. *Data catalogue*, <http://globcurrent.ifremer.fr/products-data/data-catalogue> (2013).
12. Carrasco, A., Semedo, A., Isachsen, P. E., Christensen, K. H. & Saetra, Ø. Global surface wave drift climate from ERA-40: the contributions from wind-sea and swell. *Ocean Dynamics* **64** (2014).
13. Khalaf, H. K. *The theoretical investigation of drag coefficient and settling velocity correlations* Master thesis, Nahrain University, (2009).
14. Egger, M., Sulu-Gambari, F. & Lebreton, L. First evidence of plastic fallout from the North Pacific Garbage Patch. *Scientific Reports* **10** (2020).
15. Pabortsava, K. & Lampitt, R. S. High concentrations of plastic hidden beneath the surface of the Atlantic Ocean. *Nature Communications* **11** (2020).
16. China Plastic. *China plastics industry yearbook*. (China Petrocheical Press, 2014).
17. Kooi, M., Van Nes, E. H., Scheffer, M. & Koelmans, A. A. Ups and downs in the ocean: Effects of biofouling on the vertical transport of microplastics. *Environmental Science & Technology* **51**, 7963–7971 (2017).
18. Law, K. L. *et al.* Distribution of surface plastic debris in the eastern Pacific Ocean from an 11-Year data set. *Environmental Science & Technology* **48**, 4732-4738 (2014).
19. Eriksen, M. *et al.* Plastic pollution in the world's oceans: More than 5 trillion plastic pieces weighing over 250,000 tons afloat at sea. *PloS one* **9**, e111913 (2014).
20. Law, K. L. *et al.* Plastic Accumulation in the North Atlantic Subtropical Gyre. *Science* **329**, 1185-1188 (2010).

293
